# Supplementary material for: Promoter Specificity and Transcription Levels Modulate Trans-Splicing Efficiency at the mod(mdg4) Locus in Drosophila
Source: Int J Mol Sci. 2025 Nov 29;26(23):11609. doi: 10.3390/ijms262311609 (PMC12692577; doi:10.3390/ijms262311609)
Supplement: Supplementary file 1 [file ijms-26-11609-s001.zip › ijms-3992003-supplementary.pdf]

Supplementary Materials and Methods

Model system for detecting trans-splicing at the protein level

In addition to the above-described model, we utilized a previously established *trans*-splicing model system [15]. This system assesses trans-splicing events at the protein level (Supplementary Figure 1). Briefly, it employs the same donor construct; and an acceptor construct containing a promoter-driven 3′ exon encoding the V isoform, fused to firefly luciferase. Upon trans-splicing in transgenic flies, the IRES initiates cap-independent translation of the *Fluc* reporter. Cap-dependent translation of *Fluc* is blocked due to the presence of a premature open reading frame (ORF), which is removed by trans-splicing. Previously, transgenic lines expressing intron 4 variants with deletions demonstrated a strong correlation between Fluc luminescence and RT-qPCR quantification. However, in this study, this model served an auxiliary function, as the structure of the donor portion of the chimeric transcript differed considerably, which apparently influenced translation efficiency (it is known that different untranslated regions and the presence of introns affect translation).

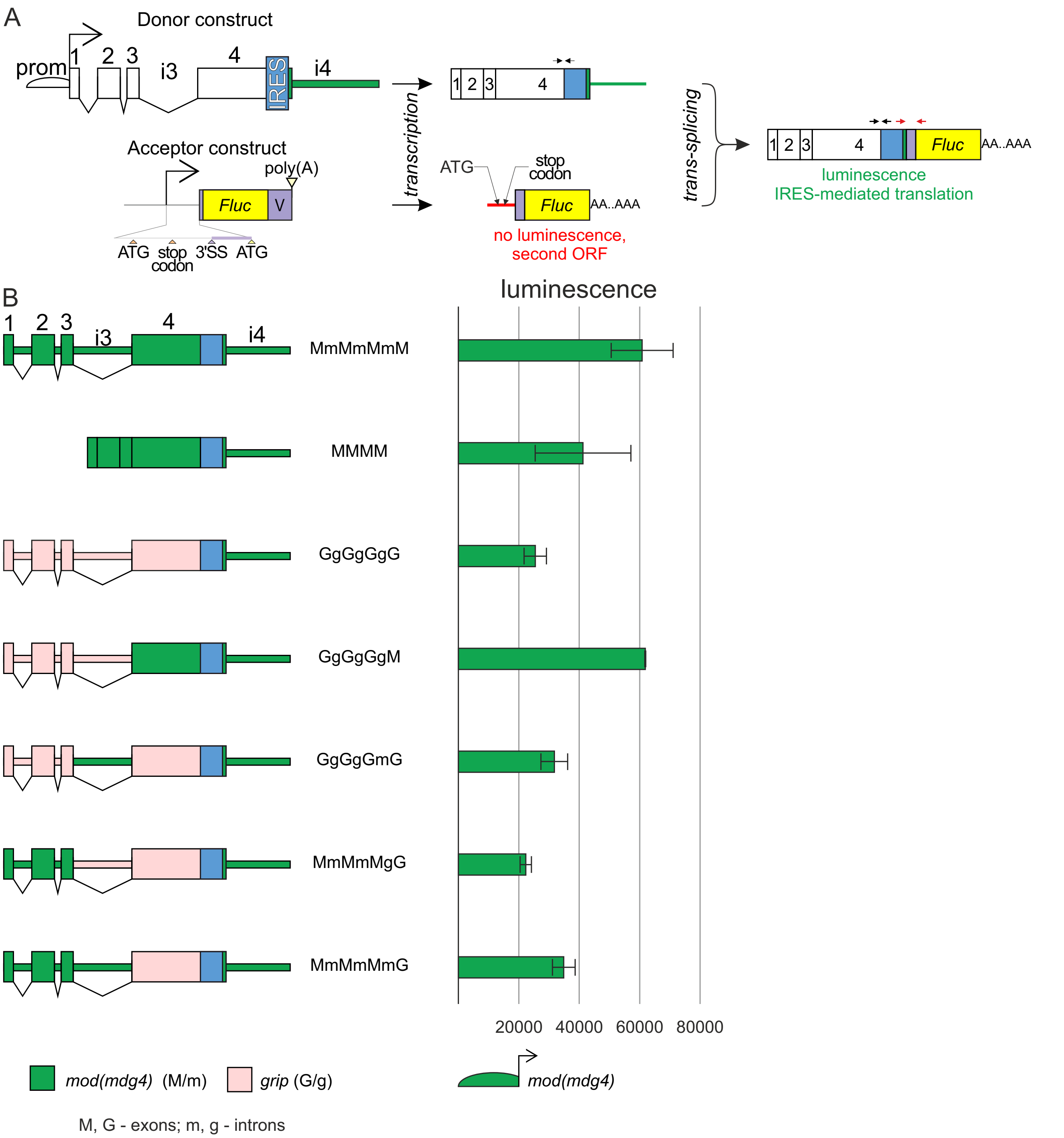

Supplementary Figure S1. Investigation of the roles of promoter, exons, and introns in *mod(mdg4) trans*-splicing. (**A**)Schematic of the model system employed to delineate regions critical for *trans*-splicing. The donor construct (top) comprises a promoter (denoted by an arrow and semicircle), constitutive exons (rectangles), introns (white lines), an internal ribosome entry site (IRES) sequence, and intron 4. The acceptor construct (bo om) includes the promoter (arrow) and alternative exon V with the *Fluc* reporter gene (yellow box). Transcription generates donor and acceptor transcripts. *Fluc* translation is obstructed by a premature open reading frame (ORF). *Trans*-splicing subsequently yields an mRNA product. In the spliced mRNA, *Fluc* can be translated through the IRES. (**B**) Schematic depictions of the donor constructs utilized to investigate the contributions of *mod(mdg4)* gene elements are presented on the left. Fragments derived from *mod(mdg4)* are colored green, while those substituted from the *grip* gene are pink; the IRES is depicted in light blue. Construct nomenclature adheres to the following scheme: uppercase le ers indicate exons, lowercase le ers signify introns, M/m denotes *mod(mdg4)*,and G/g signifies *grip*.On the right, luminescence levels are shown. Donor constructs were placed under the transcriptional control of the *mod(mdg4)* promoter.

Supplementary Table S1. List of primers used in this study cloning

name	sequence	cloning method
	**transgenic model system - donor**	
prom_fw	5'-CGAGGCTGTCATATGCTTAG-3'	bluent
exon 4 rev	5'-ACGATGATTCACTGTAGTTCTGGG-3'	bluent
IRES_fw	5'-AGGCGGCGTGAGAGAACCAGGTATA-3'	bluent
IRES_rev	5'-CCTGATCGGGTATGTAGAATGCCAC-3'	bluent
intron 4 fw	5'-CAACAGTCCCAGAACTACAG-3'	Sal I
intron 4 rev	5'-TGAGCTCCGTATCTGCGAGA-3'	Sac I
grip fw	5'-GATGCTAGCTGTATAAGACCGGTCGCATC-3'	Nhe I
grip rev	5'-GGGCTGCAGGAATTCGATTCAGGACGATCCGCTTGATCGT-3'	Pst I
ex4 Nh_r	5'-GCGGCTAGCCAGGATCGTTCTGAAAAA-3'	Nhe I
ex4 Nh_fw	5'-GCGGCTAGCAACGATCCTGCGCCTCAG-3'	Nhe I
grip ex10 Nh_r	5'-GCGGCTAGCGTTGTATGACCTGAAAGG-3'	Nhe I
grip ex10 Nh_fw	5'-GCGGCTAGCGTCATACAACCTTGGGTA-3'	Nhe I
ex3-grip i3_fw	5'-CGGATGTGAGTATTGGTTTTTGG-3'	fusion PCR
ex3-grip i3_r	5'-AACCAATACTCACATCCGTTAGC-3'	fusion PCR
grip ex9-in3_fw	5'-TCGCGGTGAGTATATGTATACAT-3'	fusion PCR
grip ex9-in3_r	5'-TACATATACTCACCGCGACGTGC-3'	fusion PCR
i3-grip e10_fw	5'-TTTTTTTTTCAGGTCATACAACCT-3'	fusion PCR
i3-grip e10_r	5'-TATGACCTGAAAAAAAAATATTGG-3'	fusion PCR
grip ex7 Nh fw	5'-GATGCTAGCTGTATAAGACCGGTCGCATC-3'	Nhe I
grip ex10 IRES r	5'-GGGCTGCAGGAATTCGATTCAGGACGATCCGCTTGATCGT-3'	fusion PCR
	**transgenic model system - acceptor**	
prom TK fw	5'-GCGCCATGGAGTTGCAGATGCGGATGTGG-3'	NcoI (Fluc)
prom TK r	5'-GCGGAATTCCCACGCGGAATATCCCCG-3'	EcoRI (mCherry)
	**genotyping**	
	**transgenic model system**	
attB fw	5'-GTCGACGATGTAGGTCACGG-3'	
loc22 rev	5'-ACCTCGGAATGAAGGAGTGAAG-3'	
	**quantitative PCR**	
	**trans-splicing**	
ex4 3' fw	5'-GGATCAGGGCAACACAGAG-3'	
Fluc r	5'-CGCCGGGCCTTTCTTTAT-3'	
mCherry r	5'-AAGCGCATGAACTCCTTGA-3'	
splT probe	5'-FAM-TCAAGCCGCCACATCCGCAT-BHQ1-3'	
splK probe	5'-FAM-CCCGATGAACTGACCTTGAGC-BHQ1-3'	
	**donor construct transcription**	
ex4tr fw	5'-CAACAGTCCCAGAACTACAG-3'	
grip fw	5'-CCACGCTGACGATCAAG-3'	
IRES r	5'-TGTTCTGGTGTCTCTCTTATTC-3'	
	**normalization**	
Vha100 fw	5'-TCATCTTCCACAACGCTTAC-3'	
Vha100 r	5'-GGAGATCCTGTTCCTGAAATAC-3'	
CG9067 fw	5'-GGAGCAGGAACTGGAATTG-3'	
CG9067 r	5'-TGTGGTTCTCTGTCGAGTAG-3'	

Supplementary Materials. Plasmid Sequences.

>M-MmMmMmM_(pSK_wlx-ex32-29rpr-MI-Rex26-attB)

GTAAAACGACGGCCAGTGAATTGTAATACGACTCACTATAGGGCGAATTGGGTACCGGGCCCCCCCTCGAGATCCCCGGGCGAGCTCGAATTAATTCTAG
TATGTATGTAAGTTAATAAAACCCTTTTTTGGAGAATGTAGATTTAAAAAAACATATTTTTTTTTATTTTTTACTGCACTGGATATCATTGAACTTATC
TGATCAGTTTTAAATTTACTTCGATCCAAGGGTATTTGAAGTACCAGGTTCTTTCGATTACCTCTCACTCAAAATGACATTCCACTCAAAGTCAGCGCTG
TTTGCCTCCTTCTCTGTCCACAGAAATATCGCCGTCTCTTTCGCCGCTGCGTCCGCTATCTCTTTCGCCACCGTTTGTAGCGTTACCTAGCGTCAATGTC
CGCCTTCAGTTGCACTTTGTCAGCGGTTTCGTGACGAAGCTCCAAGCGGTTTACGCCATCAATTAAACACAAAGTGCTGTGCCAAAACTCCTCTCGCTTC
TTATTTTTGTTTGTTTTTTGAGTGATTGGGGTGGTGATTGGTTTTGGGTGGGTAAGCAGGGGAAAGTGTGAAAAATCCCGGCAATGGGCCAAGAGGATCA
GGAGCTATTAATTCGCGGAGGCAGCAAACACCCATCTGCCGAGCATCTGAACAATGTGAGTAGTACATGTGCATACATCTTAAGTTCACTTGATCTATAG
GAACTGCGATTGCAACATCAAATTGTCTGCGGCGTGAGAACTGCGACCCACAAAAATCCCAAACCGCAATCGCACAAACAAATAGTGACACGAAACAGAT
TATTCTGGTAGCTGTGCTCGCTATATAAGACAATTTTTAAGATCATATCATGATCAAGACATCTAAAGGCATTCATTTTCGACTACATTCTTTTTTACAA
AAAATATAACAACCAGATATTTTAAGCTGATCCTAGATGCACAAAAAATAAATAAAAGTATAAACCTACTTCGTAGGATACTTCGTTTTGTTCGGGGTTA
GATGAGCATAACGCTTGTAGTTGATATTTGAGATCCCCTATCATTGCAGGGTGACAGCGGACGCTTCGCAGAGCTGCATTAACCAGGGCTTCGGGCAGGC
CAAAAACTACGGCACGCTCCTGCCACCCAGTCCGCCGGAGGACTCCGGTTCAGGGAGCGGCCAACTAGCCGAGAACCTCACCTATGCCTGGCACAATATG
GACATCTTTGGGGCGGTCAATCAGCCGGGCTCCGGATGGCGGCAGCTGGTCAACCGGACACGCGGACTATTCTGCAACGAGCGACACATACCGGCGCCCA
GGAAACATTTGCTCAAGAACGGTGAGTTTCTATTCGCAGTCGGCTGATCTGTGTGAAATCTTAATAAAGGGTCCAATTACCAATTTGAAACTCAGTTTGC
GGCGTGGCCTATCCGGGCGAACTTTTGGCCGTGATGGGCAGTTCCGGTGCCGGAAAGACGACCCTGCTGAATGCCCTTGCCTTTCGATCGCCGCAGGGCA
TCCAAGTATCGCCATCCGGGATGCGACTGCTCAATGGCCAACCTGTGGACGCCAAGGAGATGCAGGCCAGGTGCGCCTATGTCCAGCAGGATGACCTCTT
TATCGGCTCCCTAACGGCCAGGGAACACCTGATTTTCCAGGCCATGGTGCGGATGCCACGACATCTGACCTATCGGCAGCGAGTGGCCCGCGTGGATCAG
GTGATCCAGGAGCTTTCGCTCAGCAAATGTCAGCACACGATCATCGGTGTGCCCGGCAGGGTGAAAGGTCTGTCCGGCGGAGAAAGGAAGCGTCTGGCAT
TCGCCTCCGAGGCACTAACCGATCCGCCGCTTCTGATCTGCGATGAGCCCACCTCCGGACTGGACTCATTTACCGCCCACAGCGTCGTCCAGGTGCTGAA
GAAGCTGTCGCAGAAGGGCAAGACCGTCATCCTGACCATTCATCAGCCGTCTTCCGAGCTGTTTGAGCTCTTTGACAAGATCCTTCTGATGGCCGAGGGC
AGGGTAGCTTTCTTGGGCACTCCCAGCGAAGCCGTCGACTTCTTTTCCTAGTGAGTTCGATGTGTTTATTAAGGGTATCTAGCATTACATTACATCTCAA
CTCCTATCCAGCGTGGGTGCCCAGTGTCCTACCAACTACAATCCGGCGGACTTTTACGTACAGGTGTTGGCCGTTGTGCCCGGACGGGAGATCGAGTCCC
GTGATCGGATCGCCAAGATATGGCGACAATTTTGCTATTAGCAAAGTAGCCCGGGATATGGAGCAGTTGTTGGCCACCAAAAATTTGGAGAAGCCACTGG
AGCAGCCGGAGAATGGGTACACCTACAAGGCCACCTGGTTCATGCAGTTCCGGGCGGTCCTGTGGCGATCCTGGCTGTCGGTGCTCAAGGAACCACTCCT
CGTAAAAGTGCGACTTATTCAGACAACGGTGAGTGGTTCCAGTGGAAACAAATGATATAACGCTTACAATTCTTGGAAACAAATTCGCTAGATTTTAGTT
AGAATTGCCTGATTCCACACCCTTCTTAGTTTTTTTCAATGAGATGTATAGTTTATAGTTTTGCAGAAAATAAATAAATTTCATTTAACTCGCGAACATG
TTGAAGATATGAATATTAATGAGATGCGAGTAACATTTTAATTTGCAGATGGTTGCCATCTTGATTGGCCTCATCTTTTTGGGCCAACAACTCACGCAAG
TGGGCGTGATGAATATCAACGGAGCCATCTTCCTCTTCCTGACCAACATGACCTTTCAAAACGTCTTTGCCACGATAAATGTAAGTCTTGTTTAGAATAC
ATTTGCATATTAATAATTTACTAACTTTCTAATGAATCGATTCGATTTAGGTGTTCACCTCAGAGCTGCCAGTTTTTATGAGGGAGGCCCGAAGTCGACT
TTATCGCTGTGACACATACTTTCTGGGCAAAACGATTGCCGAATTACCGCTTTTTCTCACAGTGCCACTGGTCTTCACGGCGATTGCCTATCCGATGATC
GGACTGCGGGCCGGAGTGCTGCACTTCTTCAACTGCCTGGCGCTGGTCACTCTGGTGGCCAATGTGTCAACGTCCTTCGGATATCTAATATCCTGCGCCA
GCTCCTCGACCTCGATGGCGCTGTCTGTGGGTCCGCCGGTTATCATACCATTCCTGCTCTTTGGCGGCTTCTTCTTGAACTCGGGCTCGGTGCCAGTATA
CCTCAAATGGTTGTCGTACCTCTCATGGTTCCGTTACGCCAACGAGGGTCTGCTGATTAACCAATGGGCGGACGTGGAGCCGGGCGAAATTAGCTGCACA
TCGTCGAACACCACGTGCCCCAGTTCGGGCAAGGTCATCCTGGAGACGCTTAACTTCTCCGCCGCCGATCTGCCGCTGGACTACGTGGGTCTGGCCATTC
TCATCGTGAGCTTCCGGGTGCTCGCATATCTGGCTCTAAGACTTCGGGCCCGACGCAAGGAGTAGCCGACATATATCCGAAATAACTGCTTGTTTTTTT
TTTACCATTATTACCATCGTGTTTACTGTTTATTGCCCCCTCAAAAAGCTAATGTAATTATATTTGTGCCAATAAAAACAAGATATGACCTATAGGAATT
TCGAGGTCGATCGACGGTATCGCCGGGGATCTATAACTTCGTATAATGTATGCTATACGAAGTTATAAGATCCTCTAGCGATAAGCTTGATCGAGGCTGT
CATATGCTTAGGAGATTTATAAAATATGTATCCAAATATAAAAAACTAATTTATGTATTACATTTGTAAATTTAATCTAATAGCTTAAGAATATATATGG
TATTGACAATATATTTTAAGTTCTGTCCATCACTAATTCCAAACATGTATGCTTGTATGCATGTATGAAAGTATCTTTGCACAATCGCACACATTGTACC
TGACTATCAGATTCAATAACCGACTCCTCCAGCTGCTCCCCGTTGTTATTAGTTGTTTGTTCGCAGCCACTTTATGCTGATTTTGTTTCGCTACTTGAGC
TTAAGTATCCGTATTTATTATCGTAAAGTCCTTAAGTTTTATAGTCCCCATGTATTTACGTTTGTCCGCGCGTCTTGTGTATTAAACGGTTGTCAAGTGG
CGCTCAAGTGCAGATTTTCGCACACTTGATTTTAAGAGTGTACTAAGCTAACAAATAAATAAATCGTGGGGAATATGTTTGGTTTAAACGTTACGCTCTC
ATATATTATTGTTTATTTCACTTGAAACTGTTATGATTTAATAGAGATGAATAACATGGATATGTGAAGTTGTCTAAATAGAGCAACATTTTTAATAATA
TTTCGCCTTTATGGGATGAATACCTTCATTTCGAATCTTGGCATTGCACCAAAAACGTGTTCACATATGTATATAATTGCATATGTAGTGCATATGTACA
TACAGTACACAGATATACATATTATGTACATACATATTTTGTTTGGTATGTTTAAGAACTTTTAGCGAATATTTGCCTTTCTTATAATCCTATTAAGTCA
CTTTTTTTTTAAAAATATAGTGCCCTTGTGCATTCAGAGTGTAGGGTTTACGTAGTGACGATGCGCTCCATAAGAGTTTGTCAGAGTTATAATGAATTCC
GCTAGAAAATAATCCTCCATTCGTAAAAAATCGAAGGCCCAGTTTAAAAAATAGGAGAATTTAAAAAAGTTAAAATTTTTCTGTTTATCTTATGAATTTT
ATTCTAGTTGTGCTAGTTTGACTGCTAGTCTTTAAACTCGTTAATTTGTATTTTGTTTTATTTCATTTAAAATAAATTAAACATTAAGCTAGACAACAAA
ACTGTTGTGAAATAAGAAATTTCGTCATAACATAAATACATCGTATACTTAGGCCGATATTTTCTTAGTCCTACAACCTTTTTTAACACACCCGCAAACT
AAAATCCTACAGCATATGTAAACTAAAGTATGTAAAAGTTTTCAAAAAGTTATGAACTTAATTATGTGATTTTCGTACTAACATTTAAAAAAAAAATATC
AAAGCACATCAACTATCAATATTCATAAAAGTATTTCGATATAAATATTTTAAGCTATCGATATATTGTCACTTTTTGTTTTGCCATCGCCAGCAATCTC
GTCGCCTGGTAACACTTCTAATTTTCACGTCAAAGAACTCGGACGCGTTCTGCGTGTCGGCCGCGCTAGCAAAAAACTCTGGCTTTAGTTAGTTATTTTA
TTGGAAAAATATTTAGTCAAGAGGTGAGTAATGGCGCCCCAAGTGAATATACACAGTAGGTTGCGGTCATTAAGCGAGATAAATGTAATTTACACACACG
CACACACAGACAGACGCGTGAAAATTTCCCAGTTTTTTTTGGCTGCAGTGCATTCTATTTTGTGTGTGCCTTTGCCATTTTTCAGCACTCGCTGCATTGA
GTTTTAAACGCGCATTGCACTTTTTCGCCTGCAGCCAACAAACGCATAGATACAGAAAAGTATTGATTTTCGTCCAAGATGGCGGACGACGAGCAATTCA
GCTTGTGCTGGAACAACTTCAACACGAATTTGTCGGCCGGCTTCCACGAGTCGCTATGCCGCGGCGACCTGGTGGACGTCTCGCTGGCCGCCGAGGGCCA
AATAGTGAAGGCCCACCGATTGGTGTTATCCGTCTGCTCGCCCTTCTTCCGCAAGATGTTCACTCAGATGCCGTCGAACACCCACGCTATCGGTGAGTTT
GTGCCCCTCAGCTCAGATCTGTAGCTCTACGTGATTTTTTACCTTATACTCTCTTCGCCTATCTTGCAGTATTCCTGAACAACGTCAGCCACTCGGCGCT
GAAGGACCTCATCCAATTCATGTACTGCGGCGAGGTCAACGTGAAGCAGGACGCCCTGCCCGCGTTTATTAGCACCGCGGAATCGCTGCAAATCAAGGGG
CTAACGGATGTGAGTATATGTATACATTTGTGTGCGTGATATACTGGTATATCGAATCGAGCAACTACAACGCAACCTTTCGCGCCCAATACATACAGAC
GCGATGGCTGGCGTCTGTATAAGAGCATGGTTTGACTGTACCGAGAAATTTCTCGCAAACCAAATGCCAAAAGGCACGCGTGCCAGAGAGAGACAACTGC
AGGGGCTATACGCGAAGCGTTTGTCTCGCTCTGGCTCGCACGGCAGCGGCAAATTGGAGTGGTTGTTTTTGTCTCGCAGCGAATTCGATGCCAAAGCATA
AACATTTTTTGCTTTTAAACTAATGTTTAAATTGGGAAATATCTCTTAATGTTTTCGACTAAAATTAACGTATTTGCAAAATTAAATTTAGTGCAGCTGT
ACCTAAAGAAACAGGCATCGGAACTAGAAAATGTATTATTGTTTCCCAATTCAAGGAAGGCGTACTTTACTAATTAATGGCCAGATTTCTTATTTCTTTG
CAAATTCCATTCTAACGGAATAATTCTTTTCAACATATAGTTTTGAAATCGTCCATTTTATTAAATGCAGATACTTTTAAAGTTTATTAAGTTTTGATCG
TGGTTCATTTTTAAAGAGGTGCTAGAACATACAAAATATCTTTTCACTTATTCCTAAAATATATGTTAATAATTAAAAAACCAATATTTTTTTTTCAGAA
CGATCCTGCGCCTCAGCCGCCGCAGGAATCCAGCCCGCCGCCAGCAGCACCTCATGTACAACAACAGCAAATACCGGCTCAGCGGGTGCAGCGCCAGCAG
CCTCGCGCCTCCGCCCGCTACAAGATCGAGACCGTGGACGACGGCCTGGGCGACGAGAAACAGAGCACCACCCAGATCGTCATCCAGACCACGGCCGCAC
CCCAAGCCACCATTGTACAACAGCAGCAACCGCAGCAGGCGGCGCAGCAGATCCAATCGCAGCAGCTGCAGACGGGAACCACGACGACGGCGACGCTGGT
GTCGACAAACAAACGCTCTGCCCAGCGCTCCTCCCTGACCCCCGCCTCCTCGAGCGCCGGCGTAAAGCGCTCCAAGACAAGCACCTCCGCAAATGTTATG

GACCCTCTCGACTCGACCACTGAAACTGGAGCCACGACAACCGCCCAACTTGTGCCGCAACAGATCACCGTGCAAACCTCTGTGGTCTCTGCGGCAGAAG
CAAAGCTCCACCAGCAATCGCCGCAGCAGGTTCGACAGGAGGAAGCCGAGTACATTGATCTCCCCATGGAATTGCCCACGAAATCTGAACCTGACTACTC
TGAGGACCATGGCGACGCCGCCGGCGATGCCGAGGGCACCTATGTGGAGGACGATACTTACGGAGACATGCGCTACGACGACAGCTACTTCACTGAAAAC
GAGGATGCTGGCAACCAGACTGCGGCAAATACGAGCGGTGGCGGAGTGACGGCGACCACTTCCAAGGCTGTGGTCAAGCAACAGTCCCAGAACTACAGTG
AATCATCGTGAATCGAATTCCTGCAGCCCAATTCGATAGGCGGCGTGAGAGAACCAGGTATAAATAGCCTCGGAGCACTACTCAATATATTCAGTTGAAA
AGCAACCATCGCAGCGAACGCATCTCGAAAAAATCATTGAATAAGAGAGACACCAGAACAAAGTGAACGAACTCGAAAATACGAAAGAAAAGTGTGTGCG
CCAGCAACAAAGAACTAACTCGATAAATATTCATTGTGCAGAAGAGAAAGTTATTGAGTCACTACCAGTTGTGTAATTCGAACGAGAAGAAAGATAAAC
CAACAATGGCAGTGGCATTCTACATACCCGATCAGGATCAAGCTGGGGGATCCACTAGTCGAGGTCGACACCAGCGGGGATCAGGGCAACACAGAGGCTC
AAGGTTGGTTGAATTGCAAAGCATACATGGCCAGAGGCCAATAGATATAAGCAATATTTAAAGGACATTAAGTCCGCTTGCGAAATGCAAACACGTTAAA
AAAAAGATCACATATGTATTCCAAACTTGAACACTTTCCAAATCTATTAGCGTCGCAAGCGGATCATGTCCTTTTAGAGTTCGGCAAAGTGCGTTTAATT
TTGTTTTGAACAAACTTAAGCTATACTTCGCTGGCCATTAGTTAATAAGTAGTTTACGGGCCTGTGTTCTATTTCGAATCGACAAAGAGCACTAGGCAGA
TCTGCTGTCCGATGCATATTTGTTAAATGCTGGACGGAGATCGCGCGGTTTTACACTTATTCAGTTAAAAGGTAAGTAGAGTGATGAAATTTTAAGAAGC
TGCTACGATGGATATCTGATGTCAACGTGAAGCCGAACCCGTGAAGAGCTGCAACTACTCAAGGTAATACCGGCACTCTTAAAACCACACCTATCCATAT
ATTTTTCCAAAATTTGCAGGCATTTGTAATTGTTTATTTGACTGAGCAAAGCGAGTAATTGGAGTTTTCAATCTTACATGATCAGGGGGTTATTATTTGG
GCATGCGCATTTCTGTTTAATAATGATCCTCTGGTGATGCTAAGGCTACTGAGGTTTGTTGTTATTTATGTTTTCAAACTCGTACTTAAAGATGTTGTCT
GGATTTGTTTAAGCAACGTAAAAGCTTGCTGATGCAAATCCAGCAACGGCATTGATTGCCGAGTCTTAAAAAAAAAATTGTATTTATAACGCTGTGTTAT
AGTAAAGGTTCATTTCTAATCGTTTTAGTGGTCACGTAAAAAAAAAAGGAAAGATTTTAATAATACGTATAGGGAGACTCTTTTAACGTTATCAGTGGAG
AGGAAATTTATTTCTTCATGCAGCGTCGATTCTGTTTTACTTTTTGCGTGCTGATTTTGTTTTTTCATTCCAAATTTTTTGTATGGAACATTTTGCGTGT
TTTTTTTATATGGTTCCGAATTAAAGGGTGTCTGAAGAATTTCACGATATTTACGGATGTCGCGGTAACCTTAAACAAAAAAAACAGAATATACTAGACC
TCTTACCTAATTGGTAAGAGATAATCATAAACCGTTTTAATTCATATAGGCGATAGTTTATATACCTTGGAACAAAAGGTTGGACCGCCTTGAAGAATTC
CCGACTTATATTTCCTTTATTTGTAGTAGTAATTTTCGTTTTTTTATTGTTAAGCGTATTTGAGAAAATTTATATTAACGTTTGAATCGTGTAATTTATA
TTTTGCGCGCCTGGTCCGGATATTTAGAAAGTATCCACAGAATATATTATATATATATTTGTAGTATGAAAAAATAAAGAATTAAAATTTGAATTAATGC
GGAGTTGCCATTTTGTGGAAATTGAGGACGGTGTTGCCACTTCCGGAAACCCTAATTGAATGTGCAGCCCTGGTGCTTCGGCTGTGTGGCAACGCCGTGC
AAATTCATTAAAAAAATGCAAGCCAATTTGTTTTGTTATTTTATTGGATCCGAATTACACGGCCTATTTGTTCACAATTAATTATTTCCAGCTTGTGACG
GTGTGTTTGGTGTGGTGTTTATTTAATATTTAAAGTGATGGGGCTGGTAAGGGGGGCAACGCCTCCCTATTTCCACCCTTTTGATAATCTGTGATAGTGC
TTATTTGAAAACCAAGCGATGCAAATGCTACCGTAAGTTGTAAAATGCCATCCCGCCGCATTTTGGTAAGTTTTCTACCACCAAACCCAAGGGTCCTCCG
GTTCACTGATTGCGAATAAACTGCTTAAAGCCAAAGTCTAAACTAAACGTTGTTTTATTTCGCCGATCCTAACTTAACGCTTAAGCCATATATGTACATT
CTCTAGAATAGCAACTAGATCATCTGTCAATTATATTATGCCCACTAAACCCCATGCTTTCGGTTTTCCTCGACTCTCGCAGATACGGAGATCAGTTTCA
TTGGCCGCCACCGCGGTGGCTAGTTCTAGGGCCGCTCTAGCCACCATGACTTCGAAAGTTTATGATCCAGAACAAAGGAAACGGATGATAACTGGTCCGC
AGTGGTGGGCCAGATGTAAACAAATGAATGTTCTTGATTCATTTATTAATTATTATGATTCAGAAAAACATGCAGAAAATGCTGTTATTTTTTTACATGG
TAACGCGGCCTCTTCTTATTTATGGCGACATGTTGTGCCACATATTGAGCCAGTAGCGCGGTGTATTATACCAGACCTTATTGGTATGGGCAAATCAGGC
AAATCTGGTAATGGTTCTTATAGGTTACTTGATCATTACAAATATCTTACTGCATGGTTTGAACTTCTTAATTTACCAAAGAAGATCATTTTTGTCGGCC
ATGATTGGGGTGCTTGTTTGGCATTTCATTATAGCTATGAGCATCAAGATAAGATCAAAGCAATAGTTCACGCTGAAAGTGTAGTAGATGTGATTGAATC
ATGGGATGAATGGCCTGATATTGAAGAAGATATTGCGTTGATCAAATCTGAAGAAGGAGAAAAAATGGTTTTGGAGAATAACTTCTTCGTGGAAACCATG
TTGCCATCAAAAATCATGAGAAAGTTAGAACCAGAAGAATTTGCAGCATATCTTGAACCATTCAAAGAGAAAGGTGAAGTTCGTCGTCCAACATTATCAT
GGCCTCGTGAAATCCCGTTAGTAAAAGGTGGTAAACCTGACGTTGTACAAATTGTTAGGAATTATAATGCTTATCTACGTGCAAGTGATGATTTACCAAA
AATGTTTATTGAATCGGACCCAGGATTCTTTTCCAATGCTATTGTTGAAGGTGCCAAGAAGTTTCCTAATACTGAATTTGTCAAAGTAAAAGGTCTTCAT
TTTTCGCAAGAAGATGCACCTGATGAAATGGGAAAATATATCAAATCGTTCGTTGAGCGAGTTCTCAAAAATGAACAATAATTCTAGAGCGGCCAATTCT
GCAGGAATTCGATCGCAGCCAGAAGAAGAACGCCCAGCTGGTGTTCCGCAACTACATCTACAACAAGAAGCTCACCCAGGCCAATGGACAGACGACCTGG
CGCTGTGCGGATGTGCTCAAGCTGCGCTGCAAGGCGGTGGTCATCACCCGCGACGGACACTTCATCGACGCACGACGTCAGCACAATCACGAGTCCCACG
CCTCGAGGATTGGCCAGCGACAACTGTATAAGGTGGAGCAGGAACTGGAGGAGTACATCGAGATCTGCACGTCGAACCCAAAGATATCACAGTATCTGGG
CAGTAGCAACATCATTGTGACTGCCAAGGACGGCAAGGACTGCAAGTTGTTCCTGCCCGCTGCCGAGGCTACCGAGATCGAGATGCAGGCCCTTGTGGAC
GCCGCTGAGGAGGAGTTAGATGAGGAGGAGCGGCATGCAGAGGAGCGGATCCGGGATCGGCAACGGGTGGGCAGGTGGCGGACAGAAGAGGCCAAGCATC
GATCCCTGCTGAAGAGCGAGCACCCCTAGGAGTTATCAGCTAACACCTAAGCTAATTATTTATCTCGAACCACGGAAGGAGTCGATTTCACTTGGGAGTT
TTTAACCAATCTACAGTCAATCAGTTGGAAAGTCTTTATAAAGATAAGAAAGCCATAGCTGGTATTATTTTGGCTTATTTTTAAAACCTAAAACTTTCCA
GCTCTGTAATAATTAATACATATTTATTTTAATAGTAATACATCTTTATATGTTAGTAAGTCATGCAAAAATTCACAATTCCACAAGGCTCAAGACCATT
TTGCCCAAAGAAATCGACCCGACCACTCGAAAGTAGATTTTAAGCGACTTGTCTGAGTTCTGTAAACGTATTTCATACTTAGCTCCTAATAAATTAAGAC
CAAAACAAAAGATGGAGCTCATTGTGGACTGGGTAACCCAATTGTTATTAACCAAATATCTTAAGTGTAGACAACTGAACTAACGATAAATCAAGCTCTA
GAGCGGCCGTCGACGATGTAGGTCACGGTCTCGAAGCCGCGGTGCGGGTGCCAGGGCGTGCCCTTGGGCTCCCCGGGCGCGTACTCCACCTCACCCATCT
GGTCCATCATGATGAACGGGTCGAGGTGGCGGTAGTTGATCCCGGCGAACGCGCGGCGCACCGGGAAGCCCTCGCCCTCGAAACCGCTGGGCGCGGTGGT
CACGGTGAGCACGGGACGTGCGACGGCGTCGGCGGGTGCGGATACGCGGGGCAGCGTCAGCGGGTTCTCGACGGTCACGGCGGGCATGTCGACGGCCGCC
ACCGCGGTGGAGCTCCAGCTTTTGTTCCCTTTAGTGAGGGTTAATTTCGAGCTTGGCGTAATCATGGTCATAGCTGTTTCCTGTGTGAAATTGTTATCCG
CTCACAATTCCACACAACATACGAGCCGGAAGCATAAAGTGTAAAGCCTGGGGTGCCTAATGAGTGAGCTAACTCACATTAATTGCGTTGCGCTCACTGC
CCGCTTTCCAGTCGGGAAACCTGTCGTGCCAGCTGCATTAATGAATCGGCCAACGCGCGGGGAGAGGCGGTTTGCGTATTGGGCGCTCTTCCGCTTCCTC
GCTCACTGACTCGCTGCGCTCGGTCGTTCGGCTGCGGCGAGCGGTATCAGCTCACTCAAAGGCGGTAATACGGTTATCCACAGAATCAGGGGATAACGCA
GGAAAGAACATGTGAGCAAAAGGCCAGCAAAAGGCCAGGAACCGTAAAAAGGCCGCGTTGCTGGCGTTTTTCCATAGGCTCCGCCCCCCTGACGAGCATC
ACAAAAATCGACGCTCAAGTCAGAGGTGGCGAAACCCGACAGGACTATAAAGATACCAGGCGTTTCCCCCTGGAAGCTCCCTCGTGCGCTCTCCTGTTCC
GACCCTGCCGCTTACCGGATACCTGTCCGCCTTTCTCCCTTCGGGAAGCGTGGCGCTTTCTCATAGCTCACGCTGTAGGTATCTCAGTTCGGTGTAGGTC
GTTCGCTCCAAGCTGGGCTGTGTGCACGAACCCCCCGTTCAGCCCGACCGCTGCGCCTTATCCGGTAACTATCGTCTTGAGTCCAACCCGGTAAGACACG
ACTTATCGCCACTGGCAGCAGCCACTGGTAACAGGATTAGCAGAGCGAGGTATGTAGGCGGTGCTACAGAGTTCTTGAAGTGGTGGCCTAACTACGGCTA
CACTAGAAGGACAGTATTTGGTATCTGCGCTCTGCTGAAGCCAGTTACCTTCGGAAAAAGAGTTGGTAGCTCTTGATCCGGCAAACAAACCACCGCTGGT
AGCGGTGGTTTTTTTGTTTGCAAGCAGCAGATTACGCGCAGAAAAAAAGGATCTCAAGAAGATCCTTTGATCTTTTCTACGGGGTCTGACGCTCAGTGGA
ACGAAAACTCACGTTAAGGGATTTTGGTCATGAGATTATCAAAAAGGATCTTCACCTAGATCCTTTTAAATTAAAAATGAAGTTTTAAATCAATCTAAAG
TATATATGAGTAAACTTGGTCTGACAGTTACCAATGCTTAATCAGTGAGGCACCTATCTCAGCGATCTGTCTATTTCGTTCATCCATAGTTGCCTGACTC
CCCGTCGTGTAGATAACTACGATACGGGAGGGCTTACCATCTGGCCCCAGTGCTGCAATGATACCGCGAGACCCACGCTCACCGGCTCCAGATTTATCAG
CAATAAACCAGCCAGCCGGAAGGGCCGAGCGCAGAAGTGGTCCTGCAACTTTATCCGCCTCCATCCAGTCTATTAATTGTTGCCGGGAAGCTAGAGTAAG
TAGTTCGCCAGTTAATAGTTTGCGCAACGTTGTTGCCATTGCTACAGGCATCGTGGTGTCACGCTCGTCGTTTGGTATGGCTTCATTCAGCTCCGGTTCC
CAACGATCAAGGCGAGTTACATGATCCCCCATGTTGTGCAAAAAAGCGGTTAGCTCCTTCGGTCCTCCGATCGTTGTCAGAAGTAAGTTGGCCGCAGTGT
TATCACTCATGGTTATGGCAGCACTGCATAATTCTCTTACTGTCATGCCATCCGTAAGATGCTTTTCTGTGACTGGTGAGTACTCAACCAAGTCATTCTG
AGAATAGTGTATGCGGCGACCGAGTTGCTCTTGCCCGGCGTCAATACGGGATAATACCGCGCCACATAGCAGAACTTTAAAAGTGCTCATCATTGGAAAA
CGTTCTTCGGGGCGAAAACTCTCAAGGATCTTACCGCTGTTGAGATCCAGTTCGATGTAACCCACTCGTGCACCCAACTGATCTTCAGCATCTTTTACTT
TCACCAGCGTTTCTGGGTGAGCAAAAACAGGAAGGCAAAATGCCGCAAAAAAGGGAATAAGGGCGACACGGAAATGTTGAATACTCATACTCTTCCTTTT
TCAATATTATTGAAGCATTTATCAGGGTTATTGTCTCATGAGCGGATACATATTTGAATGTATTTAGAAAAATAAACAAATAGGGGTTCCGCGCACATTT
CCCCGAAAAGTGCCACCTGACGCGCCCTGTAGCGGCGCATTAAGCGCGGCGGGTGTGGTGGTTACGCGCAGCGTGACCGCTACACTTGCCAGCGCCCTAG

CGCCCGCTCCTTTCGCTTTCTTCCCTTCCTTTCTCGCCACGTTCGCCGGCTTTCCCCGTCAAGCTCTAAATCGGGGGCTCCCTTTAGGGTTCCGATTTAG
TGCTTTACGGCACCTCGACCCCAAAAAACTTGATTAGGGTGATGGTTCACGTAGTGGGCCATCGCCCTGATAGACGGTTTTTCGCCCTTTGACGTTGGAG
TCCACGTTCTTTAATAGTGGACTCTTGTTCCAAACTGGAACAACACTCAACCCTATCTCGGTCTATTCTTTTGATTTATAAGGGATTTTGCCGATTTCGG
CCTATTGGTTAAAAAATGAGCTGATTTAACAAAAATTTAACGCGAATTTTAACAAAATATTAACGCTTACAATTTCCATTCGCCATTCAGGCTGCGCAAC
TGTTGGGAAGGGCGATCGGTGCGGGCCTCTTCGCTATTACGCCAGCTGGCGAAAGGGGGATGTGCTGCAAGGCGATTAAGTTGGGTAACGCCAGGGTTTT
CCCAGTCACGACGTT

>A-GgGgGgG_(pSK_wlx-pAc-grip-rpr-MI-Rex26-attB)
GTAAAACGACGGCCAGTGAATTGTAATACGACTCACTATAGGGCGAATTGGGTACCGGGCCCCCCCTCGAGATCCCCGGGCGAGCTCGAATTAATTCTAG
TATGTATGTAAGTTAATAAAACCCTTTTTTGGAGAATGTAGATTTAAAAAAACATATTTTTTTTTTATTTTTTACTGCACTGGATATCATTGAACTTATC
TGATCAGTTTTAAATTTACTTCGATCCAAGGGTATTTGAAGTACCAGGTTCTTTCGATTACCTCTCACTCAAAATGACATTCCACTCAAAGTCAGCGCTG
TTTGCCTCCTTCTCTGTCCACAGAAATATCGCCGTCTCTTTCGCCGCTGCGTCCGCTATCTCTTTCGCCACCGTTTGTAGCGTTACCTAGCGTCAATGTC
CGCCTTCAGTTGCACTTTGTCAGCGGTTTCGTGACGAAGCTCCAAGCGGTTTACGCCATCAATTAAACACAAAGTGCTGTGCCAAAACTCCTCTCGCTTC
TTATTTTTGTTTGTTTTTTGAGTGATTGGGGTGGTGATTGGTTTTGGGTGGGTAAGCAGGGGAAAGTGTGAAAAATCCCGGCAATGGGCCAAGAGGATCA
GGAGCTATTAATTCGCGGAGGCAGCAAACACCCATCTGCCGAGCATCTGAACAATGTGAGTAGTACATGTGCATACATCTTAAGTTCACTTGATCTATAG
GAACTGCGATTGCAACATCAAATTGTCTGCGGCGTGAGAACTGCGACCCACAAAAATCCCAAACCGCAATCGCACAAACAAATAGTGACACGAAACAGAT
TATTCTGGTAGCTGTGCTCGCTATATAAGACAATTTTTAAGATCATATCATGATCAAGACATCTAAAGGCATTCATTTTCGACTACATTCTTTTTTACAA
AAAATATAACAACCAGATATTTTAAGCTGATCCTAGATGCACAAAAAATAAATAAAAGTATAAACCTACTTCGTAGGATACTTCGTTTTGTTCGGGGTTA
GATGAGCATAACGCTTGTAGTTGATATTTGAGATCCCCTATCATTGCAGGGTGACAGCGGACGCTTCGCAGAGCTGCATTAACCAGGGCTTCGGGCAGGC
CAAAAACTACGGCACGCTCCTGCCACCCAGTCCGCCGGAGGACTCCGGTTCAGGGAGCGGCCAACTAGCCGAGAACCTCACCTATGCCTGGCACAATATG
GACATCTTTGGGGCGGTCAATCAGCCGGGCTCCGGATGGCGGCAGCTGGTCAACCGGACACGCGGACTATTCTGCAACGAGCGACACATACCGGCGCCCA
GGAAACATTTGCTCAAGAACGGTGAGTTTCTATTCGCAGTCGGCTGATCTGTGTGAAATCTTAATAAAGGGTCCAATTACCAATTTGAAACTCAGTTTGC
GGCGTGGCCTATCCGGGCGAACTTTTGGCCGTGATGGGCAGTTCCGGTGCCGGAAAGACGACCCTGCTGAATGCCCTTGCCTTTCGATCGCCGCAGGGCA
TCCAAGTATCGCCATCCGGGATGCGACTGCTCAATGGCCAACCTGTGGACGCCAAGGAGATGCAGGCCAGGTGCGCCTATGTCCAGCAGGATGACCTCTT
TATCGGCTCCCTAACGGCCAGGGAACACCTGATTTTCCAGGCCATGGTGCGGATGCCACGACATCTGACCTATCGGCAGCGAGTGGCCCGCGTGGATCAG
GTGATCCAGGAGCTTTCGCTCAGCAAATGTCAGCACACGATCATCGGTGTGCCCGGCAGGGTGAAAGGTCTGTCCGGCGGAGAAAGGAAGCGTCTGGCAT
TCGCCTCCGAGGCACTAACCGATCCGCCGCTTCTGATCTGCGATGAGCCCACCTCCGGACTGGACTCATTTACCGCCCACAGCGTCGTCCAGGTGCTGAA
GAAGCTGTCGCAGAAGGGCAAGACCGTCATCCTGACCATTCATCAGCCGTCTTCCGAGCTGTTTGAGCTCTTTGACAAGATCCTTCTGATGGCCGAGGGC
AGGGTAGCTTTCTTGGGCACTCCCAGCGAAGCCGTCGACTTCTTTTCCTAGTGAGTTCGATGTGTTTATTAAGGGTATCTAGCATTACATTACATCTCAA
CTCCTATCCAGCGTGGGTGCCCAGTGTCCTACCAACTACAATCCGGCGGACTTTTACGTACAGGTGTTGGCCGTTGTGCCCGGACGGGAGATCGAGTCCC
GTGATCGGATCGCCAAGATATGGCGACAATTTTGCTATTAGCAAAGTAGCCCGGGATATGGAGCAGTTGTTGGCCACCAAAAATTTGGAGAAGCCACTGG
AGCAGCCGGAGAATGGGTACACCTACAAGGCCACCTGGTTCATGCAGTTCCGGGCGGTCCTGTGGCGATCCTGGCTGTCGGTGCTCAAGGAACCACTCCT
CGTAAAAGTGCGACTTATTCAGACAACGGTGAGTGGTTCCAGTGGAAACAAATGATATAACGCTTACAATTCTTGGAAACAAATTCGCTAGATTTTAGTT
AGAATTGCCTGATTCCACACCCTTCTTAGTTTTTTTCAATGAGATGTATAGTTTATAGTTTTGCAGAAAATAAATAAATTTCATTTAACTCGCGAACATG
TTGAAGATATGAATATTAATGAGATGCGAGTAACATTTTAATTTGCAGATGGTTGCCATCTTGATTGGCCTCATCTTTTTGGGCCAACAACTCACGCAAG
TGGGCGTGATGAATATCAACGGAGCCATCTTCCTCTTCCTGACCAACATGACCTTTCAAAACGTCTTTGCCACGATAAATGTAAGTCTTGTTTAGAATAC
ATTTGCATATTAATAATTTACTAACTTTCTAATGAATCGATTCGATTTAGGTGTTCACCTCAGAGCTGCCAGTTTTTATGAGGGAGGCCCGAAGTCGACT
TTATCGCTGTGACACATACTTTCTGGGCAAAACGATTGCCGAATTACCGCTTTTTCTCACAGTGCCACTGGTCTTCACGGCGATTGCCTATCCGATGATC
GGACTGCGGGCCGGAGTGCTGCACTTCTTCAACTGCCTGGCGCTGGTCACTCTGGTGGCCAATGTGTCAACGTCCTTCGGATATCTAATATCCTGCGCCA
GCTCCTCGACCTCGATGGCGCTGTCTGTGGGTCCGCCGGTTATCATACCATTCCTGCTCTTTGGCGGCTTCTTCTTGAACTCGGGCTCGGTGCCAGTATA
CCTCAAATGGTTGTCGTACCTCTCATGGTTCCGTTACGCCAACGAGGGTCTGCTGATTAACCAATGGGCGGACGTGGAGCCGGGCGAAATTAGCTGCACA
TCGTCGAACACCACGTGCCCCAGTTCGGGCAAGGTCATCCTGGAGACGCTTAACTTCTCCGCCGCCGATCTGCCGCTGGACTACGTGGGTCTGGCCATTC
TCATCGTGAGCTTCCGGGTGCTCGCATATCTGGCTCTAAGACTTCGGGCCCGACGCAAGGAGTAGCCGACATATATCCGAAATAACTGCTTGTTTTTTTT
TTTACCATTATTACCATCGTGTTTACTGTTTATTGCCCCCTCAAAAAGCTAATGTAATTATATTTGTGCCAATAAAAACAAGATATGACCTATAGGAATT
TCGAGGTCGATCGACGGTATCGCCGGGGATCTATAACTTCGTATAATGTATGCTATACGAAGTTATAAGATCCTCTAGCGATAAGCTCATGAATGGCATC
AACTCTGAATCAAATCTTTGCAGATGCACCTACTTCTCATTTCCACTGTCACATCATTTTTCCAGATCTCGCTGCCTGTTATGTGGCCCACAAACCAAGA
CACGTTTTATGGCCATTAAAGCTGGCTGATCGTCGCCAAACACCAAATACATATCAATATGTACATTCGAGAAAGAAGCGATCAAAGAAGCGTCTTCGGG
CGAGTAGGAGAATGCGGAGGAGAAGGAGAACGAGCTGATCTAGTATCTCTCCACAATCCAATGCCAACTGACCAACTGGCCATATTCGGAGCAATTTGAA
GCCAATTTCCATCGCCTGGCGATCGCTCCATTCTTGGCTATATGTTTTTCACCGTTCCCGGGGCCATTTTCAAAGACTCGTCGGTAAGATAAGATTGTGT
CACTCGCTGTCTCTCTTCATTTGTCGAAGAATGCTGAGGAATTTCGCGATGACGTCGGCGAGTATTTTGAAGAATGAGAATAATTTGTATTTATACGAAA
ATCAGTTAGTGGAATTTTCTACAAAAACATGTTATCTATAGATAATTTTGTTGCAAAATATGTTGACTATGACAAAGATTGTATGTATATACCTTTAATG
TATTCTCATTTTCTTATGTATTTATAATGGCAATGATGATACTGATGATATTTTAAGATGATGCCAGACCACAGGCTGATTTCTGCGTCTTTTGCCGAAC
GCAGTGCATGTGCGGTTGTTGTTTTTTGGAATAGTTTCAATTTTCGGACTGTCCGCTTTGATTTCAGTTTCTTGGCTTATTCAAAAAGCAAAGTAAAGCC
AAAAAAGCGAGATGGCAATACCAAATGCGGCAAAACGGTAGTGGAAGGAAAGGGGTGCGGGGCAGCGGAAGGAAGGGTGGGGCGGGGCGTGGCGGGGTCT
GTGGCTGGGCGCGACGTCACCGACGTTGGAGCCACTCCTTTGACCATGTGTGCGTGTGTGTATTATTCGTGTCTCGCCACTCGCCGGTTGTTTTTTTCTT
TTTATCTCGCTCTCTCTAGCGCCATCTCGTACGCATGCTCAACGCACCGCATGTTGCCGTGTCCTTTATGCGTCATTTTGGCTCGAAATAGGCAATTATT
TAAACAAAGATTAGTCAACGAAAACGCTAAAATAAATAAGTCTACAATATGGTTACTTATTGCCATGTGTGTGCAGCCAACGATAGCAACAAAAGCAACA
ACACAGTGGCTTTCCCTCTTTCACTTTTTGTTTGCAAGCGCGTGCGAGCAAGACGGCACGACCGGCAAACGCAATTACGCTGACAAAGAGCAGACGAAGT
TTTGGCCGAAAAACATCAAGGCGCCTGATACGAATGCATTTGCAATAACAATTGCGATATTTAATATTGTTTATGAAGCTGTTTGACTTCAAAACACACA
AAAAAAAAAATAAAACAAATTATTTGAAAGAGAATTAGGAATCGGACAGCTTATCGTTACGGGCTAACAGCACACCGAGACGAAATAGCTTACCTGACGT
CACAGCCTCTGGAAGAACTGCCGCCAAGCAGAGAGAGAGAGAAAAAGAGGGAGAGCAGCTTAGACCGCATGTGCTTGTGTGTGAGGCGTCTCTCTCTTCG
TCTCCTGTTTGCGCAAACGCATAGACTGCACTGAGAAAATCGATTACCTATTTTTTATGAATGAATATTTGCACTATTACTATTCAAAACTATTAAGATA
GCAATCACATTCAATAGCCAAATACTATACCACCTGAGCGATGCAACGAAATGATCAATTTGAGCAAAAATGCTGCATATTTAGGACGGCATCATTATAG
AAATGCTTCTTGCTGTGTACTTTTCTCTCGTCTGGCAGCTGTTTCGCCGTTATTGTTAAAACCGGCTTAAGTTAGGTGTGTTTTCTACGACTAGTGATGC
CCCTACTAGAAGATGTGTGTTGCACAAATGTCCCTGAATAACCAATTTGAAGTGCAGATAGCAGTAAACGTAAGCTAATATGAATATTATTTAACTGTAA
TGTTTTAATATCGCTGGACATTACTAATAAACCCACTATAAACACATGTACATATGTATGTTTTGGCATACAATGAGTAGTTGGGGAAAAAAATGTGTAAA
AGCACCGTGACCATCACAGCATAAAGATAACCAGCTGAAGTATCGAATATGAGTAACCCCCAAATTGAATCACATGCCGCAACTGATAGGACCCATGGAA
GTACACTCTTCATGGCGATATACAAGACACACACAAGCACGAACACCCAGTTGCGGAGGAAATTCTCCGTAAATGAAAACCCAATCGGCGAACAATTCAT
ACCCATATATGGTAAAAGTTTTGAACGCGACTTGAGAGCGGAGAGCATTGCGGCTGATAAGGTTTTAGCGCTAAGCGGGCTTTATAAAACGGGCTGCGGG
ACCAGTTTTCATATCACTACCGTTTGAGTTCTTGTGCTGTGTGGATACTCCTCCCGACACAAAGCCGCTCCATCAGCCAGCAGTCGTCTAATCCAGAGAC
CCCGGATCGGGTCGAGTCTAGCTGTATAAGACCGGTCGCATCAAGCCAGGCGATCGGTTGCTGCGCGTCGATAATGTGAGTGTTTTCGATTTCGAGTGAC
ACTTCCGTTTCCGCTTTGGCTTTCTCCAGAAAATTGCCAGGCCAAAAATAGTTTTTAAATATTACTACTCAATATTTTCGTTAGAATAGGTTTCGAAATA
GATCGTCTTTAATATATTTTTTCCACACAGGTCTCACTGATTGGCAAGACGCTGGCGGAGGCGCAGCAGATCATCAAGTGCGGCGGTCATGTTTCCGGCT
ACACCAACCTGACCATCGAGTACGATGTTTCTGTGGTGCAGAGCGTTGAGTTCTCGATGGGACCGCTGCTCATCGAGATCGAGCGACCGATGAACGACAA

GTTGGGCCTGGTGCTGTGCAATTATACGGCGGCTGTGCCCGCTTCCGGTTCCGGTTCCAGTACTCCTTCCAGCGGGGAGAAGATCGAGGAATCGGCAGGC
GTCTTTATCGCCAGTATCCTGCCCGCCAGCATTGCAGATCGGTAGGTTGTATATCTTCGATTTTGTTAGAATCCATTCTCATGTGCCCGTTTTCGTTTTT
GGCTTGTGTTCCAGTTGCGGCGCCTTATCCGTTGGCGATCAGGTGCTCTCCATCGACGACACAATGATCGAGCATACCGCCTTCAGTCCCGACGAGGTGA
TGACCATTTTGGACACCAGCACGGGTCGCGGCTACACGCAGATGCAGATCATGCCCGCTCACGCGCTGGCACGTCGCGGTGAGTATTGGTTTTTGGTTTG
CAAGATCCCCACATCTTCGTCTCCCATTCCTTTATTTTCGGCTATCTGGCAGTCGAAATCTGCAAGGCCAAATCAGCCAGGCTCCCAAGCGGCGCGACGA
AGGTCTGCGTGGGCGTCACGGCTAATGAACAGCAAAAGCATCGGCGGCTGAAACAATAGAGACGCCAGGCCTTATAAAGTTTGGAGCGAGGAGAGATCCA
TGAGTTCCAGATGAAGTTACTAGTCAGAAACTAAAGTTAAAGTTACAATATTATACAATTTAAGACTCAGTATACGGCAGATTATAGTATTGTTTCCTAC
CATAAGTTCAGTTCTCCACTTGATATCGTCATATATTTTCTTCCAAGAACCGTTCCATCCACCCTCGCTGCAGCCAATCAATTGTTTTTCCAAGTTTTTC
CCTCCATTTGCTAAGCAAACACGCAGCGACGCAGTCCAAAATCGTTTCCCCCAAGACGAAAGACGGAATAGTCTCTTTTTTTTTTTGACAATCGATCGAA
GCCGAATCGGAAATAGCAAATGGAATAGAAAATAACAGAGGAGAGTGGAGGCCATAAATTGTGGTCTGGGCAGAGAGGACACGCAGCTGGCTAAGCTGCG
GATCAGTCGCCAGAGTGGAGTCTTCGGCACAAGGCCAACATAGCCATCGGAGAAGGTCTTAAAAGCTTCCATAAGTTGGCAGGGTGGGAGGTGTGATTGA
ATGTATCAGATATTCAAGGGGGAAATTACTTAGCCAAATGGAAAGAGAGAAGGAAGCGGAGGAGGAGGAGGCACCACTCCCACTCGCTTTTGTAGTGGGG
GGATCTATGAATCTTGTATACAATATGCTTAATAAACCCTTGGTACTAGAAGAAATTCATGAGACGTTATATCTAATCCTTTCAGGTCATACAACCTTGG
GTAGTCCCAAGTACAGCTTTAGCACACTGGAGTCCCGTAAATCCTCGACGGCGGGTCGCCAGCGTCAGCGTTTCGCGAGGAAAAGTTCCCTACCGTTGGA
GAATCCTGCATGCGGCACTCCAGGCTCCACGGGAGGATCGATGGGTACTGGTGGCATCCATGGCAGCAGCAGCTTGGGCATGGGTCTGGGCCTCTGCCGG
GCGGAGAGCTTTCCCGTTCTCCTCGATTGCAGCCATGGAGCGGGCATTATCTTGTCTGAAACGGTATCCGGATCTGGAGGATCGGGTGCTGTAGCCATTG
CTCAGATCGTGCCGGATTCGGTGGCCGATCGCAGTGGCTGCATTCAGGCGGGAGATCGCATCGTGGCCATTAACAAGATGTACAGCTTGGATGCGGCGGC
CATGAGGCAACTGCTCGAAGGAGGATCCAGTCGCAATGGAGCAAGCAATGGAACGCCGGCCAACTGGCTAGAACTGGAGATCGAGTTCGATATGCCGGAT
GCCGTGGTGCCCGCCAGTGGTGTCTTTAGTGTCAAATTGCTCAGGGCCGGCAAATGTGGACTGGGTCTGAGTGTGAGTGGTTCCAGTCATGGTGGTCTGG
TCATTTCAGACGTCAAAATGGGCAGTCCTGCCCATCGCAGTGGCTCCCTTCGATCAGGGGACATTCTGCTTGCCGTGGACCAGCATCCAGTGCAGCATTT
CAACGTGGACGCACTGCTCAAGGAGGAGGGCAGATCACAGAATCCCAGCCACAGCTCCTCGGACTTCACCACGCTGACGATCAAGCGGATCGTCCTGAAT
CGAATTCCTGCAGCCCAATTCGATAGGCGGCGTGAGAGAACCAGGTATAAATAGCCTCGGAGCACTACTCAATATATTCAGTTGAAAAGCAACCATCGCA
GCGAACGCATCTCGAAAAAATCATTGAATAAGAGAGACACCAGAACAAAGTGAACGAACTCGAAAATACGAAAGAAAAGTGTGTGCGCCAGCAACAAAGA
ACTAACTCGATAAATATTCATTGTGCAGAAGAGAAAGTTATTGAGTCACTACCAGTTGTGTAATTCCGAACGAGAAGAAAGATAAACCAACAATGGCAGT
GGCATTCTACATACCCGATCAGGATCAAGCTGGGGGATCCACTAGTCGAGGTCGACACCAGCGGGGATCAGGGCAACACAGAGGCTCAAGGTTGGTTGAA
TTGCAAAGCATACATGGCCAGAGGCCAATAGATATAAGCAATATTTAAAGGACATTAAGTCCGCTTGCGAAATGCAAACACGTTAAAAAAAAGATCACAT
ATGTATTCCAAACTTGAACACTTTCCAAATCTATTAGCGTCGCAAGCGGATCATGTCCTTTTAGAGTTCGGCAAAGTGCGTTTAATTTTGTTTTGAACAA
ACTTAAGCTATACTTCGCTGGCCATTAGTTAATAAGTAGTTTACGGGCCTGTGTTCTATTTCGAATCGACAAAGAGCACTAGGCAGATCTGCTGTCCGAT
GCATATTTGTTAAATGCTGGACGGAGATCGCGCGGTTTTACACTTATTCAGTTAAAAGGTAAGTAGAGTGATGAAATTTTAAGAAGCTGCTACGATGGAT
ATCTGATGTCAACGTGAAGCCGAACCCGTGAAGAGCTGCAACTACTCAAGGTAATACCGGCACTCTTAAAACCACACCTATCCATATATTTTTCCAAAAT
TTGCAGGCATTTGTAATTGTTTATTTGACTGAGCAAAGCGAGTAATTGGAGTTTTCAATCTTACATGATCAGGGGGTTATTATTTGGGCATGCGCATTTC
TGTTTAATAATGATCCTCTGGTGATGCTAAGGCTACTGAGGTTTGTTGTTATTTATGTTTTCAAACTCGTACTTAAAGATGTTGTCTGGATTTGTTTAAG
CAACGTAAAAGCTTGCTGATGCAAATCCAGCAACGGCATTGATTGCCGAGTCTTAAAAAAAAAATTGTATTTATAACGCTGTGTTATAGTAAAGGTTCAT
TTCTAATCGTTTTAGTGGTCACGTAAAAAAAAAAGGAAAGATTTTAATAATACGTATAGGGAGACTCTTTTAACGTTATCAGTGGAGAGGAAATTTATTT
CTTCATGCAGCGTCGATTCTGTTTTACTTTTTGCGTGCTGATTTTGTTTTTTCATTCCAAATTTTTTGTATGGAACATTTTGCGTGTTTTTTTTATATGG
TTCCGAATTAAAGGGTGTCTGAAGAATTTCACGATATTTACGGATGTCGCGGTAACCTTAAACAAAAAAAACAGAATATACTAGACCTCTTACCTAATTG
GTAAGAGATAATCATAAACCGTTTTAATTCATATAGGCGATAGTTTATATACCTTGGAACAAAAGGTTGGACCGCCTTGAAGAATTCCCGACTTATATTT
CCTTTATTTGTAGTAGTAATTTTCGTTTTTTTATTGTTAAGCGTATTTGAGAAAATTTATATTAACGTTTGAATCGTGTAATTTATATTTTGCGCGCCTG
GTCCGGATATTTAGAAAGTATCCACAGAATATATTATATATATATTTGTAGTATGAAAAAATAAAGAATTAAAATTTGAATTAATGCGGAGTTGCCATTT
TGTGGAAATTGAGGACGGTGTTGCCACTTCCGGAAACCCTAATTGAATGTGCAGCCCTGGTGCTTCGGCTGTGTGGCAACGCCGTGCAAATTCATTAAAA
AAATGCAAGCCAATTTGTTTTGTTATTTTATTGGATCCGAATTACACGGCCTATTTGTTCACAATTAATTATTTCCAGCTTGTGACGGTGTGTTTGGTGT
GGTGTTTATTTAATATTTAAAGTGATGGGGCTGGTAAGGGGGGCAACGCCTCCCTATTTCCACCCTTTTGATAATCTGTGATAGTGCTTATTTGAAAACC
AAGCGATGCAAATGCTACCGTAAGTTGTAAAATGCCATCCCGCCGCATTTTGGTAAGTTTTCTACCACCAAACCCAAGGGTCCTCCGGTTCACTGATTGC
GAATAAACTGCTTAAAGCCAAAGTCTAAACTAAACGTTGTTTTATTTCGCCGATCCTAACTTAACGCTTAAGCCATATATGTACATTCTCTAGAATAGCA
ACTAGATCATCTGTCAATTATATTATGCCCACTAAACCCCATGCTTTCGGTTTTCCTCGACTCTCGCAGATACGGAGATCAGTTTCATTGGCCGCCACCG
CGGTGGCTAGTTCTAGGGCCGCTCTAGCCACCATGACTTCGAAAGTTTATGATCCAGAACAAAGGAAACGGATGATAACTGGTCCGCAGTGGTGGGCCAG
ATGTAAACAAATGAATGTTCTTGATTCATTTATTAATTATTATGATTCAGAAAAACATGCAGAAAATGCTGTTATTTTTTTACATGGTAACGCGGCCTCT
TCTTATTTATGGCGACATGTTGTGCCACATATTGAGCCAGTAGCGCGGTGTATTATACCAGACCTTATTGGTATGGGCAAATCAGGCAAATCTGGTAATG
GTTCTTATAGGTTACTTGATCATTACAAATATCTTACTGCATGGTTTGAACTTCTTAATTTACCAAAGAAGATCATTTTTGTCGGCCATGATTGGGGTGC
TTGTTTGGCATTTCATTATAGCTATGAGCATCAAGATAAGATCAAAGCAATAGTTCACGCTGAAAGTGTAGTAGATGTGATTGAATCATGGGATGAATGG
CCTGATATTGAAGAAGATATTGCGTTGATCAAATCTGAAGAAGGAGAAAAAATGGTTTTGGAGAATAACTTCTTCGTGGAAACCATGTTGCCATCAAAAA
TCATGAGAAAGTTAGAACCAGAAGAATTTGCAGCATATCTTGAACCATTCAAAGAGAAAGGTGAAGTTCGTCGTCCAACATTATCATGGCCTCGTGAAAT
CCCGTTAGTAAAAGGTGGTAAACCTGACGTTGTACAAATTGTTAGGAATTATAATGCTTATCTACGTGCAAGTGATGATTTACCAAAAATGTTTATTGAA
TCGGACCCAGGATTCTTTTCCAATGCTATTGTTGAAGGTGCCAAGAAGTTTCCTAATACTGAATTTGTCAAAGTAAAAGGTCTTCATTTTTCGCAAGAAG
ATGCACCTGATGAAATGGGAAAATATATCAAATCGTTCGTTGAGCGAGTTCTCAAAAATGAACAATAATTCTAGAGCGGCCAATTCTGCAGGAATTCGAT
CGCAGCCAGAAGAAGAACGCCCAGCTGGTGTTCCGCAACTACATCTACAACAAGAAGCTCACCCAGGCCAATGGACAGACGACCTGGCGCTGTGCGGATG
TGCTCAAGCTGCGCTGCAAGGCGGTGGTCATCACCCGCGACGGACACTTCATCGACGCACGACGTCAGCACAATCACGAGTCCCACGCCTCGAGGATTGG
CCAGCGACAACTGTATAAGGTGGAGCAGGAACTGGAGGAGTACATCGAGATCTGCACGTCGAACCCAAAGATATCACAGTATCTGGGCAGTAGCAACATC
ATTGTGACTGCCAAGGACGGCAAGGACTGCAAGTTGTTCCTGCCCGCTGCCGAGGCTACCGAGATCGAGATGCAGGCCCTTGTGGACGCCGCTGAGGAGG
AGTTAGATGAGGAGGAGCGGCATGCAGAGGAGCGGATCCGGGATCGGCAACGGGTGGGCAGGTGGCGGACAGAAGAGGCCAAGCATCGATCCCTGCTGAA
GAGCGAGCACCCCTAGGAGTTATCAGCTAACACCTAAGCTAATTATTTATCTCGAACCACGGAAGGAGTCGATTTCACTTGGGAGTTTTTAACCAATCTA
CAGTCAATCAGTTGGAAAGTCTTTATAAAGATAAGAAAGCCATAGCTGGTATTATTTTGGCTTATTTTTAAAACCTAAAACTTTCCAGCTCTGTAATAAT
TAATACATATTTATTTTAATAGTAATACATCTTTATATGTTAGTAAGTCATGCAAAAATTCACAATTCCACAAGGCTCAAGACCATTTTGCCCAAAGAAA
TCGACCCGACCACTCGAAAGTAGATTTTAAGCGACTTGTCTGAGTTCTGTAAACGTATTTCATACTTAGCTCCTAATAAATTAAGACCAAAACAAAAGAT
GGAGCTCATTGTGGACTGGGTAACCCAATTGTTATTAACCAAATATCTTAAGTGTAGACAACTGAACTAACGATAAATCAAGCTCTAGAGCGGCCGTCGA
CGATGTAGGTCACGGTCTCGAAGCCGCGGTGCGGGTGCCAGGGCGTGCCCTTGGGCTCCCCGGGCGCGTACTCCACCTCACCCATCTGGTCCATCATGAT
GAACGGGTCGAGGTGGCGGTAGTTGATCCCGGCGAACGCGCGGCGCACCGGGAAGCCCTCGCCCTCGAAACCGCTGGGCGCGGTGGTCACGGTGAGCACG
GGACGTGCGACGGCGTCGGCGGGTGCGGATACGCGGGGCAGCGTCAGCGGGTTCTCGACGGTCACGGCGGGCATGTCGACGGCCGCCACCGCGGTGGAGC
TCCAGCTTTTGTTCCCTTTAGTGAGGGTTAATTTCGAGCTTGGCGTAATCATGGTCATAGCTGTTTCCTGTGTGAAATTGTTATCCGCTCACAATTCCAC
ACAACATACGAGCCGGAAGCATAAAGTGTAAAGCCTGGGGTGCCTAATGAGTGAGCTAACTCACATTAATTGCGTTGCGCTCACTGCCCGCTTTCCAGTC
GGGAAACCTGTCGTGCCAGCTGCATTAATGAATCGGCCAACGCGCGGGGAGAGGCGGTTTGCGTATTGGGCGCTCTTCCGCTTCCTCGCTCACTGACTCG
CTGCGCTCGGTCGTTCGGCTGCGGCGAGCGGTATCAGCTCACTCAAAGGCGGTAATACGGTTATCCACAGAATCAGGGGATAACGCAGGAAAGAACATGT
GAGCAAAAGGCCAGCAAAAGGCCAGGAACCGTAAAAAGGCCGCGTTGCTGGCGTTTTTCCATAGGCTCCGCCCCCCTGACGAGCATCACAAAAATCGACG
CTCAAGTCAGAGGTGGCGAAACCCGACAGGACTATAAAGATACCAGGCGTTTCCCCCTGGAAGCTCCCTCGTGCGCTCTCCTGTTCCGACCCTGCCGCTT

ACCGGATACCTGTCCGCCTTTCTCCCTTCGGGAAGCGTGGCGCTTTCTCATAGCTCACGCTGTAGGTATCTCAGTTCGGTGTAGGTCGTTCGCTCCAAGC
TGGGCTGTGTGCACGAACCCCCCGTTCAGCCCGACCGCTGCGCCTTATCCGGTAACTATCGTCTTGAGTCCAACCCGGTAAGACACGACTTATCGCCACT
GGCAGCAGCCACTGGTAACAGGATTAGCAGAGCGAGGTATGTAGGCGGTGCTACAGAGTTCTTGAAGTGGTGGCCTAACTACGGCTACACTAGAAGGACA
GTATTTGGTATCTGCGCTCTGCTGAAGCCAGTTACCTTCGGAAAAAGAGTTGGTAGCTCTTGATCCGGCAAACAAACCACCGCTGGTAGCGGTGGTTTTT
TTGTTTGCAAGCAGCAGATTACGCGCAGAAAAAAAGGATCTCAAGAAGATCCTTTGATCTTTTCTACGGGGTCTGACGCTCAGTGGAACGAAAACTCACG
TTAAGGGATTTTGGTCATGAGATTATCAAAAAGGATCTTCACCTAGATCCTTTTAAATTAAAAATGAAGTTTTAAATCAATCTAAAGTATATATGAGTAA
ACTTGGTCTGACAGTTACCAATGCTTAATCAGTGAGGCACCTATCTCAGCGATCTGTCTATTTCGTTCATCCATAGTTGCCTGACTCCCCGTCGTGTAGA
TAACTACGATACGGGAGGGCTTACCATCTGGCCCCAGTGCTGCAATGATACCGCGAGACCCACGCTCACCGGCTCCAGATTTATCAGCAATAAACCAGCC
AGCCGGAAGGGCCGAGCGCAGAAGTGGTCCTGCAACTTTATCCGCCTCCATCCAGTCTATTAATTGTTGCCGGGAAGCTAGAGTAAGTAGTTCGCCAGTT
AATAGTTTGCGCAACGTTGTTGCCATTGCTACAGGCATCGTGGTGTCACGCTCGTCGTTTGGTATGGCTTCATTCAGCTCCGGTTCCCAACGATCAAGGC
GAGTTACATGATCCCCCATGTTGTGCAAAAAAGCGGTTAGCTCCTTCGGTCCTCCGATCGTTGTCAGAAGTAAGTTGGCCGCAGTGTTATCACTCATGGT
TATGGCAGCACTGCATAATTCTCTTACTGTCATGCCATCCGTAAGATGCTTTTCTGTGACTGGTGAGTACTCAACCAAGTCATTCTGAGAATAGTGTATG
CGGCGACCGAGTTGCTCTTGCCCGGCGTCAATACGGGATAATACCGCGCCACATAGCAGAACTTTAAAAGTGCTCATCATTGGAAAACGTTCTTCGGGGC
GAAAACTCTCAAGGATCTTACCGCTGTTGAGATCCAGTTCGATGTAACCCACTCGTGCACCCAACTGATCTTCAGCATCTTTTACTTTCACCAGCGTTTC
TGGGTGAGCAAAAACAGGAAGGCAAAATGCCGCAAAAAAGGGAATAAGGGCGACACGGAAATGTTGAATACTCATACTCTTCCTTTTTCAATATTATTGA
AGCATTTATCAGGGTTATTGTCTCATGAGCGGATACATATTTGAATGTATTTAGAAAAATAAACAAATAGGGGTTCCGCGCACATTTCCCCGAAAAGTGC
CACCTGACGCGCCCTGTAGCGGCGCATTAAGCGCGGCGGGTGTGGTGGTTACGCGCAGCGTGACCGCTACACTTGCCAGCGCCCTAGCGCCCGCTCCTTT
CGCTTTCTTCCCTTCCTTTCTCGCCACGTTCGCCGGCTTTCCCCGTCAAGCTCTAAATCGGGGGCTCCCTTTAGGGTTCCGATTTAGTGCTTTACGGCAC
CTCGACCCCAAAAAACTTGATTAGGGTGATGGTTCACGTAGTGGGCCATCGCCCTGATAGACGGTTTTTCGCCCTTTGACGTTGGAGTCCACGTTCTTTA
ATAGTGGACTCTTGTTCCAAACTGGAACAACACTCAACCCTATCTCGGTCTATTCTTTTGATTTATAAGGGATTTTGCCGATTTCGGCCTATTGGTTAAA
AAATGAGCTGATTTAACAAAAATTTAACGCGAATTTTAACAAAATATTAACGCTTACAATTTCCATTCGCCATTCAGGCTGCGCAACTGTTGGGAAGGGC
GATCGGTGCGGGCCTCTTCGCTATTACGCCAGCTGGCGAAAGGGGGATGTGCTGCAAGGCGATTAAGTTGGGTAACGCCAGGGTTTTCCCAGTCACGACG
TT

>Acceptor_(pSK_wlx_Acc3)
GTAAAACGACGGCCAGTGAATTGTAATACGACTCACTATAGGGCGAATTGGGTACCGGGCCCCCCCTCGAGATCCCCGGGCGAGCTCGAATTAATTCTAG
TATGTATGTAAGTTAATAAAACCCTTTTTTGGAGAATGTAGATTTAAAAAAACATATTTTTTTTTTATTTTTTACTGCACTGGATATCATTGAACTTATC
TGATCAGTTTTAAATTTACTTCGATCCAAGGGTATTTGAAGTACCAGGTTCTTTCGATTACCTCTCACTCAAAATGACATTCCACTCAAAGTCAGGCTG
TTTGCCTCCTTCTCTGTCCACAGAAATATCGCCGTCTCTTTCGCCGCTGCGTCCGCTATCTCTTTCGCCACCGTTTGTAGCGTTACCTAGCGTCAATGTC
CGCCTTCAGTTGCACTTTGTCAGCGGTTTCGTGACGAAGCTCCAAGCGGTTTACGCCATCAATTAAACACAAAGTGCTGTGCCAAAACTCCTCTCGCTTC
TTATTTTTGTTTGTTTTTTGAGTGATTGGGGTGGTGATTGGTTTTGGGTGGGTAAGCAGGGGAAAGTGTGAAAAATCCCGGCAATGGGCCAAGAGGATCA
GGAGCTATTAATTCGCGGAGGCAGCAAACACCCATCTGCCGAGCATCTGAACAATGTGAGTAGTACATGTGCATACATCTTAAGTTCACTTGATCTATAG
GAACTGCGATTGCAACATCAAATTGTCTGCGGCGTGAGAACTGCGACCCACAAAAATCCCAAACCGCAATCGCACAAACAAATAGTGACACGAAACAGAT
TATTCTGGTAGCTGTGCTCGCTATATAAGACAATTTTTAAGATCATATCATGATCAAGACATCTAAAGGCATTCATTTTCGACTACATTCTTTTTTACAA
AAAATATAACAACCAGATATTTTAAGCTGATCCTAGATGCACAAAAAATAAATAAAAGTATAAACCTACTTCGTAGGATACTTCGTTTTGTTCGGGGTTA
GATGAGCATAACGCTTGTAGTTGATATTTGAGATCCCCTATCATTGCAGGGTGACAGCGGACGCTTCGCAGAGCTGCATTAACCAGGGCTTCGGGCAGGC
CAAAAACTACGGCACGCTCCTGCCACCCAGTCCGCCGGAGGACTCCGGTTCAGGGAGCGGCCAACTAGCCGAGAACCTCACCTATGCCTGGCACAATATG
GACATCTTTGGGGCGGTCAATCAGCCGGGCTCCGGATGGCGGCAGCTGGTCAACCGGACACGCGGACTATTCTGCAACGAGCGACACATACCGGCGCCCA
GGAAACATTTGCTCAAGAACGGTGAGTTTCTATTCGCAGTCGGCTGATCTGTGTGAAATCTTAATAAAGGGTCCAATTACCAATTTGAAACTCAGTTTGC
GGCGTGGCCTATCCGGGCGAACTTTTGGCCGTGATGGGCAGTTCCGGTGCCGGAAAGACGACCCTGCTGAATGCCCTTGCCTTTCGATCGCCGCAGGGCA
TCCAAGTATCGCCATCCGGGATGCGACTGCTCAATGGCCAACCTGTGGACGCCAAGGAGATGCAGGCCAGGTGCGCCTATGTCCAGCAGGATGACCTCTT
TATCGGCTCCCTAACGGCCAGGGAACACCTGATTTTCCAGGCCATGGTGCGGATGCCACGACATCTGACCTATCGGCAGCGAGTGGCCCGCGTGGATCAG
GTGATCCAGGAGCTTTCGCTCAGCAAATGTCAGCACACGATCATCGGTGTGCCCGGCAGGGTGAAAGGTCTGTCCGGCGGAGAAAGGAAGCGTCTGGCAT
TCGCCTCCGAGGCACTAACCGATCCGCCGCTTCTGATCTGCGATGAGCCCACCTCCGGACTGGACTCATTTACCGCCCACAGCGTCGTCCAGGTGCTGAA
GAAGCTGTCGCAGAAGGGCAAGACCGTCATCCTGACCATTCATCAGCCGTCTTCCGAGCTGTTTGAGCTCTTTGACAAGATCCTTCTGATGGCCGAGGGC
AGGGTAGCTTTCTTGGGCACTCCCAGCGAAGCCGTCGACTTCTTTTCCTAGTGAGTTCGATGTGTTTATTAAGGGTATCTAGCATTACATTACATCTCAA
CTCCTATCCAGCGTGGGTGCCCAGTGTCCTACCAACTACAATCCGGCGGACTTTTACGTACAGGTGTTGGCCGTTGTGCCCGGACGGGAGATCGAGTCCC
GTGATCGGATCGCCAAGATATGGCGACAATTTTGCTATTAGCAAAGTAGCCCGGGATATGGAGCAGTTGTTGGCCACCAAAAATTTGGAGAAGCCACTGG
AGCAGCCGGAGAATGGGTACACCTACAAGGCCACCTGGTTCATGCAGTTCCGGGCGGTCCTGTGGCGATCCTGGCTGTCGGTGCTCAAGGAACCACTCCT
CGTAAAAGTGCGACTTATTCAGACAACGGTGAGTGGTTCCAGTGGAAACAAATGATATAACGCTTACAATTCTTGGAAACAAATTCGCTAGATTTTAGTT
AGAATTGCCTGATTCCACACCCTTCTTAGTTTTTTTCAATGAGATGTATAGTTTATAGTTTTGCAGAAAATAAATAAATTTCATTTAACTCGCGAACATG
TTGAAGATATGAATATTAATGAGATGCGAGTAACATTTTAATTTGCAGATGGTTGCCATCTTGATTGGCCTCATCTTTTTGGGCCAACAACTCACGCAAG
TGGGCGTGATGAATATCAACGGAGCCATCTTCCTCTTCCTGACCAACATGACCTTTCAAAACGTCTTTGCCACGATAAATGTAAGTCTTGTTTAGAATAC
ATTTGCATATTAATAATTTACTAACTTTCTAATGAATCGATTCGATTTAGGTGTTCACCTCAGAGCTGCCAGTTTTTATGAGGGAGGCCCGAAGTCGACT
TTATCGCTGTGACACATACTTTCTGGGCAAAACGATTGCCGAATTACCGCTTTTTCTCACAGTGCCACTGGTCTTCACGGCGATTGCCTATCCGATGATC
GGACTGCGGGCCGGAGTGCTGCACTTCTTCAACTGCCTGGCGCTGGTCACTCTGGTGGCCAATGTGTCAACGTCCTTCGGATATCTAATATCCTGCGCCA
GCTCCTCGACCTCGATGGCGCTGTCTGTGGGTCCGCCGGTTATCATACCATTCCTGCTCTTTGGCGGCTTCTTCTTGAACTCGGGCTCGGTGCCAGTATA
CCTCAAATGGTTGTCGTACCTCTCATGGTTCCGTTACGCCAACGAGGGTCTGCTGATTAACCAATGGGCGGACGTGGAGCCGGGCGAAATTAGCTGCACA
TCGTCGAACACCACGTGCCCCAGTTCGGGCAAGGTCATCCTGGAGACGCTTAACTTCTCCGCCGCCGATCTGCCGCTGGACTACGTGGGTCTGGCCATTC
TCATCGTGAGCTTCCGGGTGCTCGCATATCTGGCTCTAAGACTTCGGGCCCGACGCAAGGAGTAGCCGACATATATCCGAAATAACTGCTTGTTTTTTTT
TTTACCATTATTACCATCGTGTTTACTGTTTATTGCCCCCTCAAAAAGCTAATGTAATTATATTTGTGCCAATAAAAACAAGATATGACCTATAGGAATT
TCGAGGTCGATCGACGGTATCGCCGGGGATCTATAACTTCGTATAATGTATGCTATACGAAGTTATAAGATCCTCTAGCGATAAGCTTCGACGGATCCTT
ATCGATTTACCACATTTGTAGAGGTTTTACTTGCTTTAAAAAACCTCCCACACCTCCCCCTGAACCTGAAACATAAAATGAATGCAATTGTTGTTGTTA
ACTTGTTTATTGCAGCTTATAATGGTTACAAATAAAGCAATAGCATCACAAATTTCACAAATAAAGCATTTTTTTCACTGCATTCTAGTTGTGGTTTGTC
CAAACTCATCAATGTATCTTATCATGTCTGCTCGAAGCGGCCGGCCGCCCCGACTCTAGAATTACACGGCGATCTTTCCGCCCTTCTTGGCCTTTATGAG
GATCTCTCTGATTTTTCTTGCGTCGAGTTTTCCGGTAAGACCTTTCGGTACTTCGTCCACAAACACAACTCCTCCGCGCAACTTTTTCGCGGTTGTTACT
TGACTGGCGACGTAATCCACGATCTCTTTTTCCGTCATCGTCTTTCCGTGCTCCAAAACAACAACGGCGGCGGGAAGTTCACCGGCGTCATCGTCGGGAA
GACCTGCGACACCTGCGTCGAAGATGTTGGGGTGTTGGAGCAAGATGGATTCCAATTCAGCGGGAGCCACCTGATAGCCTTTGTACTTAATCAGAGACTT
CAGGCGGTCAACGATGAAGAAGTGTTCGTCTTCGTCCCAGTAAGCTATGTCTCCAGAATGTAGCCATCCATCCTTGTCAATCAAGGCGTTGGTCGCTTCC
GGATTGTTTACATAACCGGACATAATCATAGGACCTCTCACACACAGTTCGCCTCTTTGATTAACGCCCAGCGTTTTCCCGGTATCCAGATCCACAACCT
TCGCTTCAAAAAATGGAACAACTTTACCGACCGCGCCCGGTTTATCATCCCCCTCGGGTGTAATCAGAATAGCTGATGTAGTCTCAGTGAGCCCATATCC
TTGCCTGATACCTGGCAGATGGAACCTCTTGGCAACCGCTTCCCCGACTTCCTTAGAGAGGGGAGCGCCACCAGAAGCAATTTCGTGTAAATTAGATAAA
TCGTATTTGTCAATCAGAGTGCTTTTGGCGAAGAAGGAGAATAGGGTTGGCACCAGCAGCGCACTTTGAATCTTGTAATCCTGAAGGCTCCTCAGAAACA
GCTCTTCTTCAAATCTATACATTAAGACGACTCGAAATCCACATATCAAATATCCGAGTGTAGTAAACATTCCAAAACCGTGATGGAATGGAACAACACT

TAAAATCGCAGTATCCGGAATGATTTGATTGCCAAAAATAGGATCTCTGGCATGCGAGAATCTCACGCAGGCAGTTCTATGAGGCAGAGCGACACCTTTA
GGCAGACCAGTAGATCCAGAGGAGTTCATGATCAGTGCAATTGTCTTGTCCCTATCGAAGGACTCTGGCACAAAATCGTATTCATTAAAACCGGGAGGTA
GATGAGATGTGACGAACGTGTACATCGACTGAAATCCCTGGTAATCCGTTTTAGAATCCATGATAATAATTTTTTGGATGATTGGGAGCTTTTTTTGCAC
GTTCAAAATTTTTTGCAACCCCTTTTTGGAAACGAACACCACGGTAGGCTGCGAAATGCCCATACTGTTGAGCAATTCACGTTCATTATAAATGTCGTTC
GCGGGCGCAACTGCAACTCCGATAAATAACGCGCCCAACACCGGCATAAAGAATTGAAGAGAGTTTTCACTGCATACGACGATTCTGTGATTTGTATTCA
GCCCATATCGTTTCATAGCTTCTGCCAACCGAACGGACATTTCGAAGTACTCAGCGTAAGTGATGTCCACCTCGATATGTGCATCTGTAAAAGCAATTGT
TCCAGGAACCAGGGCGTATCTCTTCATAGCCTTATGCAGTTGCTCTCCAGCGGTTCCATCTTCCAGCGGATAGAATGGCGCCGGGCCTTTCTTTATGTTT
TTGGCGTCTTCCATGGAGTTGCAGATGCGGATGTGGCGGCTGTCAATTGGAGAATCAAACGCAACAGGTTAGAACATCTGGGTAAGATTGGGCCTTTCGG
GCGAAAAAATTAAAATAAAACAACTGTGGAGGGTCTTTTGCTGGTTCTTGTGTATTTTATTGGTTTACAAGCAGGGAGTAATGTACAAATATATAAAATA
AAATATTTAATGCACGAAAAACACGGTTTAAAGAAGAAGAGCACTGTACTTTAGAGCTGGCACTCACTATGTTATCGATGTTTGGCACCTAACATTATCG
ATTAGTGCTTTTCTATTTGGCTAAATATGTTATTTTGCAAAATCATTTATATTTATTTATCAGTCGAAGGGATTAGCAGGAAAAAATGTGTAAGGGATCG
CTTCTGTAATATCAGAATACATGTTCTCATAGTAATAGTGCTTCTTCTTGCCTTGATACACCATCGATTGTTGAGCTATGAGCTTACATTTATGGAATTT
TTCAGATATAAGACAATGCGAATTCTGAAAATAAAAAAAAATATAGCAAGAATATCAGTAAAATATAGATATGTAAAAATATAATACGTTACGATTCAAA
CACAGTTAACAGCCGATAACCGATAGCTTCGCTAACAGCACTGCTTGTGCTGCTGTTGGCACGCCATTTTTGTTGTAGTTGTTATTTTTTCGCCGTGTTG
TGTTCGCTGCGTTTTTGTTTTTTGTCAAACAAATTGCAAGTCTATAGTTAACAAAAGGTGTTGTGGCTAACTGCAGCATGCATATTAATGTCCGAAGTTT
ACAATATGTGAATTATCCTGCGAAAATGCAGCTAGTAAGTGCGTGTGGCCCTGCGCGTATGTATGTGCGTCTGTGTGTGTGCAGCGATTGTCATCTTTGT
TGTTGTTGGTGTCTCAAAAAGGAATCCCTCCAAAAAAGTACCAAAAAGGATGATGTTCTATTTGAATTTAATGTGAACTAGTGTTTTTAGTGATATTTAG
TGATTTTTCTGCTCTGGTAGTTGTGGAATTAATGTGGAAAGTGCTTTATGCCCCAGGTGAGTCCACCTTGCTACCAATCAACCCTTCTTGACCACAAAAG
GGATGTCTAGCCAAACACTAGAATATTAGACTATCAATCGACTTGTAGCCAGTTAAAGGCAAACTAGCTACAGCCTTCTACAAGTGATAAAAATAAGCGG
CTATAAATAGTAAGAGTGTATCGGGAATTGCCTGCAATAAATAGTTTACTCAGCACCTCAAACTTCCATGTGTTTTTCTCTGGGCTATACCTTTTTATTT
TCAAACCACCCGGGTAATAAAGTCAGTTTCCTTGGATCCTCCATTACAGGTCAGTTCATCGGGGATATTCCGCGTGGGAATTCATGGTGAGCAAGGGCGA
GGAGGACAACATGGCCATCATCAAGGAGTTCATGCGCTTCAAGGTGCACATGGAGGGCTCCGTGAACGGCCACGAGTTCGAGATCGAGGGCGAGGGCGAG
GGCCGCCCCTACGAGGGCACCCAGACCGCCAAGCTGAAGGTGACCAAGGGCGGCCCCCTGCCCTTCGCCTGGGACATCCTGTCCCCTCAGTTCATGTACG
GCTCCAAGGCCTACGTGAAGCACCCCGCCGACATCCCCGACTACTTGAAGCTGTCCTTCCCCGAGGGCTTCAAGTGGGAGCGCGTGATGAACTTCGAGGA
CGGCGGCGTGGTGACCGTGACCCAGGACTCCTCCCTGCAGGACGGCGAGTTCATCTACAAGGTGAAGCTGCGCGGCACCAACTTCCCCTCCGACGGCCCC
GTAATGCAGAAGAAGACCATGGGCTGGGAGGCCTCCTCCGAGCGGATGTACCCCGAGGACGGCGCCCTGAAGGGCGAGATCAAGCAGAGGCTGAAGCTGA
AGGACGGCGGCCACTACGACGCCGAGGTCAAGACCACCTACAAGGCCAAGAAGCCCGTGCAGCTGCCCGGCGCCTACAACGTCAACATCAAGCTGGACAT
CACCTCCCACAACGAGGACTACACCATCGTGGAACAGTACGAGCGCGCCGAGGGCCGCCACTCCACCGGCGGCATGGACGAGCTGTACAAGTAAGGATCC
AGACATGATAAGATACATTGATGAGTTTGGACAAACCACAACTAGAATGCAGTGAAAAAAATGCTTTATTTGTGAAATTTGTGATGCTATTGCTTTATTT
GTAACCATTATAAGCTGCAATAAACAAGTTAACAACAACAATTGCATTCATTTTATGTTTCAGGTTCAGGGGGAGGTGTGGGAGGTTTTTTAAAGCAAGT
AAAACCTCTACAAATGTGGTATGGCTGATTATGATCAGTCGACTAGTTCTAGAGCGGCCGTCGACGATGTAGGTCACGGTCTCGAAGCCGCGGTGCGGGT
GCCAGGGCGTGCCCTTGGGCTCCCCGGGCGCGTACTCCACCTCACCCATCTGGTCCATCATGATGAACGGGTCGAGGTGGCGGTAGTTGATCCCGGCGAA
CGCGCGGCGCACCGGGAAGCCCTCGCCCTCGAAACCGCTGGGCGCGGTGGTCACGGTGAGCACGGGACGTGCGACGGCGTCGGCGGGTGCGGATACGCGG
GGCAGCGTCAGCGGGTTCTCGACGGTCACGGCGGGCATGTCGACGGCCGCCACCGCGGTGGAGCTCCAGCTTTTGTTCCCTTTAGTGAGGGTTAATTTCG
AGCTTGGCGTAATCATGGTCATAGCTGTTTCCTGTGTGAAATTGTTATCCGCTCACAATTCCACACAACATACGAGCCGGAAGCATAAAGTGTAAAGCCT
GGGGTGCCTAATGAGTGAGCTAACTCACATTAATTGCGTTGCGCTCACTGCCCGCTTTCCAGTCGGGAAACCTGTCGTGCCAGCTGCATTAATGAATCGG
CCAACGCGCGGGGAGAGGCGGTTTGCGTATTGGGCGCTCTTCCGCTTCCTCGCTCACTGACTCGCTGCGCTCGGTCGTTCGGCTGCGGCGAGCGGTATCA
GCTCACTCAAAGGCGGTAATACGGTTATCCACAGAATCAGGGGATAACGCAGGAAAGAACATGTGAGCAAAAGGCCAGCAAAAGGCCAGGAACCGTAAAA
AGGCCGCGTTGCTGGCGTTTTTCCATAGGCTCCGCCCCCCTGACGAGCATCACAAAAATCGACGCTCAAGTCAGAGGTGGCGAAACCCGACAGGACTATA
AAGATACCAGGCGTTTCCCCCTGGAAGCTCCCTCGTGCGCTCTCCTGTTCCGACCCTGCCGCTTACCGGATACCTGTCCGCCTTTCTCCCTTCGGGAAGC
GTGGCGCTTTCTCATAGCTCACGCTGTAGGTATCTCAGTTCGGTGTAGGTCGTTCGCTCCAAGCTGGGCTGTGTGCACGAACCCCCCGTTCAGCCCGACC
GCTGCGCCTTATCCGGTAACTATCGTCTTGAGTCCAACCCGGTAAGACACGACTTATCGCCACTGGCAGCAGCCACTGGTAACAGGATTAGCAGAGCGAG
GTATGTAGGCGGTGCTACAGAGTTCTTGAAGTGGTGGCCTAACTACGGCTACACTAGAAGGACAGTATTTGGTATCTGCGCTCTGCTGAAGCCAGTTACC
TTCGGAAAAAGAGTTGGTAGCTCTTGATCCGGCAAACAAACCACCGCTGGTAGCGGTGGTTTTTTTGTTTGCAAGCAGCAGATTACGCGCAGAAAAAAAG
GATCTCAAGAAGATCCTTTGATCTTTTCTACGGGGTCTGACGCTCAGTGGAACGAAAACTCACGTTAAGGGATTTTGGTCATGAGATTATCAAAAAGGAT
CTTCACCTAGATCCTTTTAAATTAAAAATGAAGTTTTAAATCAATCTAAAGTATATATGAGTAAACTTGGTCTGACAGTTACCAATGCTTAATCAGTGAG
GCACCTATCTCAGCGATCTGTCTATTTCGTTCATCCATAGTTGCCTGACTCCCCGTCGTGTAGATAACTACGATACGGGAGGGCTTACCATCTGGCCCCA
GTGCTGCAATGATACCGCGAGACCCACGCTCACCGGCTCCAGATTTATCAGCAATAAACCAGCCAGCCGGAAGGGCCGAGCGCAGAAGTGGTCCTGCAAC
TTTATCCGCCTCCATCCAGTCTATTAATTGTTGCCGGGAAGCTAGAGTAAGTAGTTCGCCAGTTAATAGTTTGCGCAACGTTGTTGCCATTGCTACAGGC
ATCGTGGTGTCACGCTCGTCGTTTGGTATGGCTTCATTCAGCTCCGGTTCCCAACGATCAAGGCGAGTTACATGATCCCCCATGTTGTGCAAAAAAGCGG
TTAGCTCCTTCGGTCCTCCGATCGTTGTCAGAAGTAAGTTGGCCGCAGTGTTATCACTCATGGTTATGGCAGCACTGCATAATTCTCTTACTGTCATGCC
ATCCGTAAGATGCTTTTCTGTGACTGGTGAGTACTCAACCAAGTCATTCTGAGAATAGTGTATGCGGCGACCGAGTTGCTCTTGCCCGGCGTCAATACGG
GATAATACCGCGCCACATAGCAGAACTTTAAAAGTGCTCATCATTGGAAAACGTTCTTCGGGGCGAAAACTCTCAAGGATCTTACCGCTGTTGAGATCCA
GTTCGATGTAACCCACTCGTGCACCCAACTGATCTTCAGCATCTTTTACTTTCACCAGCGTTTCTGGGTGAGCAAAAACAGGAAGGCAAAATGCCGCAAA
AAAGGGAATAAGGGCGACACGGAAATGTTGAATACTCATACTCTTCCTTTTTCAATATTATTGAAGCATTTATCAGGGTTATTGTCTCATGAGCGGATAC
ATATTTGAATGTATTTAGAAAAATAAACAAATAGGGGTTCCGCGCACATTTCCCCGAAAAGTGCCACCTGACGCGCCCTGTAGCGGCGCATTAAGCGCGG
CGGGTGTGGTGGTTACGCGCAGCGTGACCGCTACACTTGCCAGCGCCCTAGCGCCCGCTCCTTTCGCTTTCTTCCCTTCCTTTCTCGCCACGTTCGCCGG
CTTTCCCCGTCAAGCTCTAAATCGGGGGCTCCCTTTAGGGTTCCGATTTAGTGCTTTACGGCACCTCGACCCCAAAAAACTTGATTAGGGTGATGGTTCA
CGTAGTGGGCCATCGCCCTGATAGACGGTTTTTCGCCCTTTGACGTTGGAGTCCACGTTCTTTAATAGTGGACTCTTGTTCCAAACTGGAACAACACTCA
ACCCTATCTCGGTCTATTCTTTTGATTTATAAGGGATTTTGCCGATTTCGGCCTATTGGTTAAAAAATGAGCTGATTTAACAAAAATTTAACGCGAATTT
TAACAAAATATTAACGCTTACAATTTCCATTCGCCATTCAGGCTGCGCAACTGTTGGGAAGGGCGATCGGTGCGGGCCTCTTCGCTATTACGCCAGCTGG
CGAAAGGGGGATGTGCTGCAAGGCGATTAAGTTGGGTAACGCCAGGGTTTTCCCAGTCACGACGTT
